# Self-control depletion in tufted capuchin monkeys (*Sapajus* spp.): does delay of gratification rely on a limited resource?

**DOI:** 10.3389/fpsyg.2015.01193

**Published:** 2015-08-11

**Authors:** Francesca De Petrillo, Antonia Micucci, Emanuele Gori, Valentina Truppa, Dan Ariely, Elsa Addessi

**Affiliations:** ^1^Unità di Primatologia Cognitiva e Centro Primati, Istituto di Scienze e Tecnologie della Cognizione – Consiglio Nazionale delle RicercheRome, Italy; ^2^Dipartimento di Biologia Ambientale, Sapienza Università di RomaRome, Italy; ^3^Duke UniversityDurham, NC, USA

**Keywords:** self-control, depletion, strength model, capuchin monkeys, non-human primates

## Abstract

Self-control failure has enormous personal and societal consequences. One of the most debated models explaining why self-control breaks down is the Strength Model, according to which self-control depends on a limited resource. Either previous acts of self-control or taking part in highly demanding cognitive tasks have been shown to reduce self-control, possibly due to a reduction in blood glucose levels. However, several studies yielded negative findings, and recent meta-analyses questioned the robustness of the depletion effect in humans. We investigated, for the first time, whether the Strength Model applies to a non-human primate species, the tufted capuchin monkey. We tested five capuchins in a self-control task (the Accumulation task) in which food items were accumulated within individual’s reach for as long as the subject refrained from taking them. We evaluated whether capuchins’ performance decreases: (i) when tested before receiving their daily meal rather than after consuming it (Energy Depletion Experiment), and (ii) after being tested in two tasks with different levels of cognitive complexity (Cognitive Depletion Experiment). We also tested, in both experiments, how implementing self-control in each trial of the Accumulation task affected this capacity within each session and/or across consecutive sessions. Repeated acts of self-control in each trial of the Accumulation task progressively reduced this capacity within each session, as predicted by the Strength Model. However, neither experiencing a reduction in energy level nor taking part in a highly demanding cognitive task decreased performance in the subsequent Accumulation task. Thus, whereas capuchins seem to be vulnerable to within-session depletion effects, to other extents our findings are in line with the growing body of studies that failed to find a depletion effect in humans. Methodological issues potentially affecting the lack of depletion effects in capuchins are discussed.

## Introduction

Self-control can be defined as the ability to inhibit a dominant response in order to achieve a future goal (Mischel, 1974; Tangney et al., 2004; Osvath and Osvath, 2008), and is considered an important key to success in life for both human and non-human animals. For example, people with greater self-control often have lower caloric intake, are more conscious about their food choices and gain less weight over time. Moreover, although people can withdraw money from their interest-bearing account whenever they wish, only individuals who abstain from withdrawing too often can obtain high future benefits (e.g., Read, 2004). Similarly, when foraging, non-human animals may have to decide whether to exploit a low-quality food source encountered first, rather than moving farther to find a higher quality food source (e.g., Stevens, 2010).

Failure in self-control has enormous personal and societal consequences (e.g., Mischel et al., 1989; Gottfredson and Hirschi, 1990; Baumeister et al., 1994; Tangney et al., 2004; Vohs and Faber, 2007). For instance, when self-control was depleted by prior exertion, the frequency of dishonest and unethical behaviors increased (Mead et al., 2009; Gino et al., 2011). Several models have been proposed to explain in which circumstances self-control breaks down (for a review see Muraven et al., 1998). One of the most debated models is the Strength Model (Baumeister, 2002), according to which all acts of self-control deplete a limited resource causing short-term impairments in subsequent acts of self-control. The first empirical support to this model was presented in two studies employing a dual-task paradigm (Baumeister et al., 1998; Muraven et al., 1998). Participants were initially asked to exert either a high level or a relatively low level of self-control, and then had to complete a different task in which self-control was also required. In the first experiment of Muraven et al. (1998), participants required to suppress or amplify their emotions while watching a sad movie, subsequently showed less persistence in squeezing a handgrip than participants who had not been required to regulate their emotions while watching the movie. In a second experiment, participants forced to suppress a forbidden thought were less able to refrain from laughing in response to a comedy video clip, or quit working much sooner on a potentially frustrating unsolvable anagram than participants who had been allowed to freely express their thoughts. More recently, it has been observed that taking part in highly demanding cognitive tasks, such as the switching arithmetic task (where subjects are required to switch between addition and subtraction) may decrease performance in a subsequent self-control task (Kiesel et al., 2010; Schneider and Anderson, 2010; Barutchu et al., 2013). Thus, the above findings support the hypothesis of self-control as a limited resource that can become temporarily depleted.

Overall, the Strength Model seems to apply to multiple domains, such as eating, drinking, spending money, sexuality, decision-making, and morality (for a review see Baumeister et al., 2007). Several studies have investigated the physiological mechanism underlying self-control depletion, finding that the exertion of self-control reduces blood glucose levels (Fairclough and Houston, 2004), low levels of blood glucose after performing a self-control task may predict poor performance on a subsequent self-control task, and performance on self-control tasks improves after ingesting a glucose drink (Gailliot et al., 2007; for a review see Gailliot and Baumeister, 2007). Notably, there is recent evidence that only tasting glucose is sufficient to restore self-control, without the need of ingesting it (Molden et al., 2012; Sanders et al., 2012).

However, other studies did not support this model, finding that energy depletion does not decrease self-control performance in humans (Kurzban, 2010; Molden et al., 2012; Lange and Eggert, 2014; Lange et al., 2014). For instance, participants tested in a dual-task procedure performed similarly in a self-control task after consuming a sugary drink or a non-caloric sweet drink (Lange and Eggert, 2014; Lange et al., 2014). Moreover, it has been recently demonstrated that beliefs about willpower limitations do affect sensitivity to self-control depletion, in that only participants believing (or induced to believe) that willpower may be limited by exertion, showed self-control improvement after glucose consumption (Job et al., 2013). Furthermore, two meta-analyses of the same 198 published tests of the Strength Model yielded contrasting results. Whereas Hagger et al. (2010) concluded that the depletion effect is robust and replicable, more recently Carter and McCullough (2014) warned that, when correcting for small-study effects (such as publication bias), the evidence for the depletion effect is not convincing (for a further analysis yielding negative findings, see also Carter et al., 2015).

Since a wealth of data have demonstrated that even non-human animals are able to exert self-control (e.g., birds: Wascher et al., 2012; Auersperg et al., 2013; Hillemann et al., 2014; domestic dogs: Leonardi et al., 2012; non-human primates: Beran et al., 1999; Beran, 2002; Beran and Evans, 2006; Rosati et al., 2007; Addessi et al., 2011; Pelé et al., 2011), it is surprising that few studies have so far investigated the validity of the Strength Model in non-human animals, focusing on domestic dogs, *Canis familiaris* (Miller et al., 2010, 2015; Miller and Bender, 2012) and, very recently, honeybees, *Apis mellifera* (Mayack and Naug, 2015). Dog studies employed a dual-task procedure similar to that used with humans: subjects required to maintain the “stay” position for 10 min before manipulating an unsolvable task (a Tug-A-Jug toy that did not release any food), persisted on this potentially frustrating task for a shorter time than when they were not previously required to exert self-control. The depletion effect disappeared after offering dogs a glucose drink (Miller et al., 2010). Likewise, in a subsequent study dogs performed better in a working memory task when tested 30 min after breakfast, rather than when they were fasted. Similar differences were not observed when dogs were tested 90 min after breakfast consumption (Miller and Bender, 2012). Interestingly, a low energetic state reduced self-control in honeybees tested in a Delay choice task: after a 24-h starvation period, honeybees significantly preferred the smaller immediate option over the larger delayed option, but the same did not occur when they were tested after either 6 or 18 h of starvation (Mayack and Naug, 2015). Overall, it appears that also in the non-human animal species tested so far, self-control relies on a limited energy resource and that, in dogs, glucose level is the underlying physiological correlate of self-control depletion.

Although all the studies performed in non-human animals showed positive evidence of self-control depletion, the human literature is much more controversial. In order to evaluate the consistency of self-control depletion in non-human animals, further studies are thus needed. Since dogs, and especially honeybees, are evolutionarily very distant from humans, it appears particularly relevant to investigate, for the first time, whether the Strength Model applies to non-human primates, our closest extant relatives which, however, are devoid of typically human beliefs and cultural influences. Specifically, we tested tufted capuchin monkeys (*Sapajus* spp.^1^) which, despite more than 35 million years of independent evolution, show convergences with humans in several life-history traits (including feeding behavior, a prolonged infancy and a long lifespan; Fragaszy et al., 2004). They have also been successfully employed for investigating aspects of cognition once considered uniquely human, such as stone tool use, analogical reasoning and symbolic reasoning (e.g., Addessi et al., 2007, 2008; Visalberghi et al., 2009; Truppa et al., 2011), although they diverge from hominids in other cognition domains, such as metacognition, mirror self-recognition, perspective-taking (e.g., Hare et al., 2003; de Waal et al., 2005; Paukner et al., 2006; Beran et al., 2009). Even more importantly for the present work, capuchins’ self-control abilities have been extensively explored in several studies using different experimental paradigms (Lakshminarayanan and Santos, 2009; Anderson et al., 2010; Addessi et al., 2011, 2013; Pelé et al., 2011; Bramlett et al., 2012; Evans et al., 2012; Judge and Essler, 2013; Paglieri et al., 2013). Capuchins have shown excellent motor inhibition skills (MacLean et al., 2014) and, across tasks, different levels of self-control (*sensu* Beran, 2015). Their tolerance to delay ranged from 10 to 40 s (in different captive colonies) in the Accumulation task, a self-control task in which food items are accumulated at a fixed rate in front of the subject, but accumulation stops as soon as the subject takes one or more items; thus, to obtain the maximum possible amount of food, the subject has to refrain from taking the items already available until the end of the accumulation process (Anderson et al., 2010; Pelé et al., 2011; Evans et al., 2012; Addessi et al., 2013). Interestingly, capuchins were able to wait about 80 s in a Delay choice task in which they could choose a larger/later option over an immediate/smaller one (Addessi et al., 2011), the latter performance not being significantly different from what has been observed in great apes, our closest living relatives (Rosati et al., 2007). However, there is increasing evidence that in Delay choice tasks in which subjects are required to point at visible food items, at least some of the choices for the larger delayed option are indeed due to a failure to inhibit a prepotent response toward the larger quantity rather than to willingness to wait (Paglieri et al., 2013; Addessi et al., 2014; see also Genty et al., 2012). In support of the above hypothesis is the observation that, when the same capuchin monkey group was tested in both the Accumulation task and the Delay choice task, performances in these tasks did not significantly correlate (Addessi et al., 2013). Recently, alternative paradigms have been proposed to investigate self-control abilities in capuchins. Bramlett et al. (2012) presented capuchins with a series of choices between two differently preferred food items on a revolving tray that moved those foods sequentially toward the subject, which could take the first item or wait for the second. Most capuchins waited for a highly preferred food item or a larger amount of the same food, inhibiting the prepotent response to take the less preferred/smaller option. More mixed results have been obtained in another recent study in which, after exchanging a token corresponding to a low-value food, capuchins were provided with a choice between the low-value food associated with the token or another token associated with a high-value food. Only two capuchins out of seven correctly selected the token significantly more than expected by chance (Judge and Essler, 2013).

To evaluate whether capuchin monkeys are sensitive to self-control depletion effects, we tested five subjects in the Accumulation task (see above) in two experiments. In the Energy Depletion Experiment, we tested capuchins in the Accumulation task after half an hour from the beginning of the consumption of their daily meal (*Low Energy Depletion* condition) or immediately before they received their main meal (*High Energy Depletion* condition). In the Cognitive Depletion Experiment, we tested capuchins in the Accumulation task soon after having tested them in a non-cognitively demanding Simple Touching task, in which they had just to touch a rewarding image (RI) on a touchscreen (*Low Cognitive Depletion* condition) or in a more cognitively demanding Identity Matching-To-Sample task (*High Cognitive Depletion* condition). In both experiments, we also evaluated how implementing self-control in each trial of the Accumulation task affected this capacity within each session (within-session depletion) and/or across consecutive sessions (between-session depletion). According to the Strength Model, capuchins should show a lower capacity of delaying gratification in the high depletion conditions compared to the low depletion conditions and, in both experiments, their performance in the Accumulation task should decrease within and across sessions.

## Materials and Methods

### Ethical Statement

This study complied with protocols approved by the Italian Health Ministry (DM 123/214-C to E. Addessi and DM 132/2014-C to V. Truppa). All procedures were performed in full accordance with the Directive 2010/63/EU on the protection of animals used for scientific purposes and conformed to the “Guidelines for the treatment of animals in behavioral research and teaching” (Association for the Study of Animal Behaviour/Animal Behavior Society [ASAB/ABS], 2015).

### Subjects

Subjects were five adult capuchin monkeys, three females and two males (14–30-year-old), all born in captivity and housed at the Primate Center of the Institute of Cognitive Sciences and Technologies, CNR, Rome, Italy. They belonged to four social groups, each housed in an indoor–outdoor enclosure (indoor: 5 m^2^ × 2.5 m high; outdoor: 40–130 m^2^ × 3 m high). Each subject was separated from the group just before the daily session solely for the purpose of testing. Subjects were tested either between 10:00 and 11:00 AM (Cognitive Depletion Experiment), or between 2:00 and 3:00 PM (Energy Depletion Experiment). Sessions were administered 5 days a week. Water was available *ad libitum*. Monkeys were fed every afternoon after testing (except in the *Low Energy Depletion* condition of the Energy Depletion Experiment, see below) with fresh fruits and carrots (about 350 g per animal), lettuce (about 120 g per animal), bread (about 60 g per animal), and monkey chow (Altromin-A pellets, Rieper standard diet for primates: A. Rieper SpA, Molino/Industria Mangimi, Vandoies, BZ, about 70 g per animal). Boiled eggs and potatoes were provided two times a week, and a mixture of curd cheese, vitamins, egg, bran, oats and sugar was given once a week as supplementation. According to the Primate Info Net website (University of Wisconsin, Madison, 2015), this amount of food is required to maintain capuchins’ body weight. Monkeys were never food deprived for testing.

All subjects had been previously tested in the self-control task used in the present study (Addessi et al., 2013) and were already familiar with the computerized apparatus used in the Cognitive Depletion Experiment (see below) because they took part in previous studies involving computerized Matching-to-Sample and two-alternative choice tasks (e.g., Truppa et al., 2010, 2011, 2014, 2015).

### Experimental Apparatus

**FIGURE 1:**
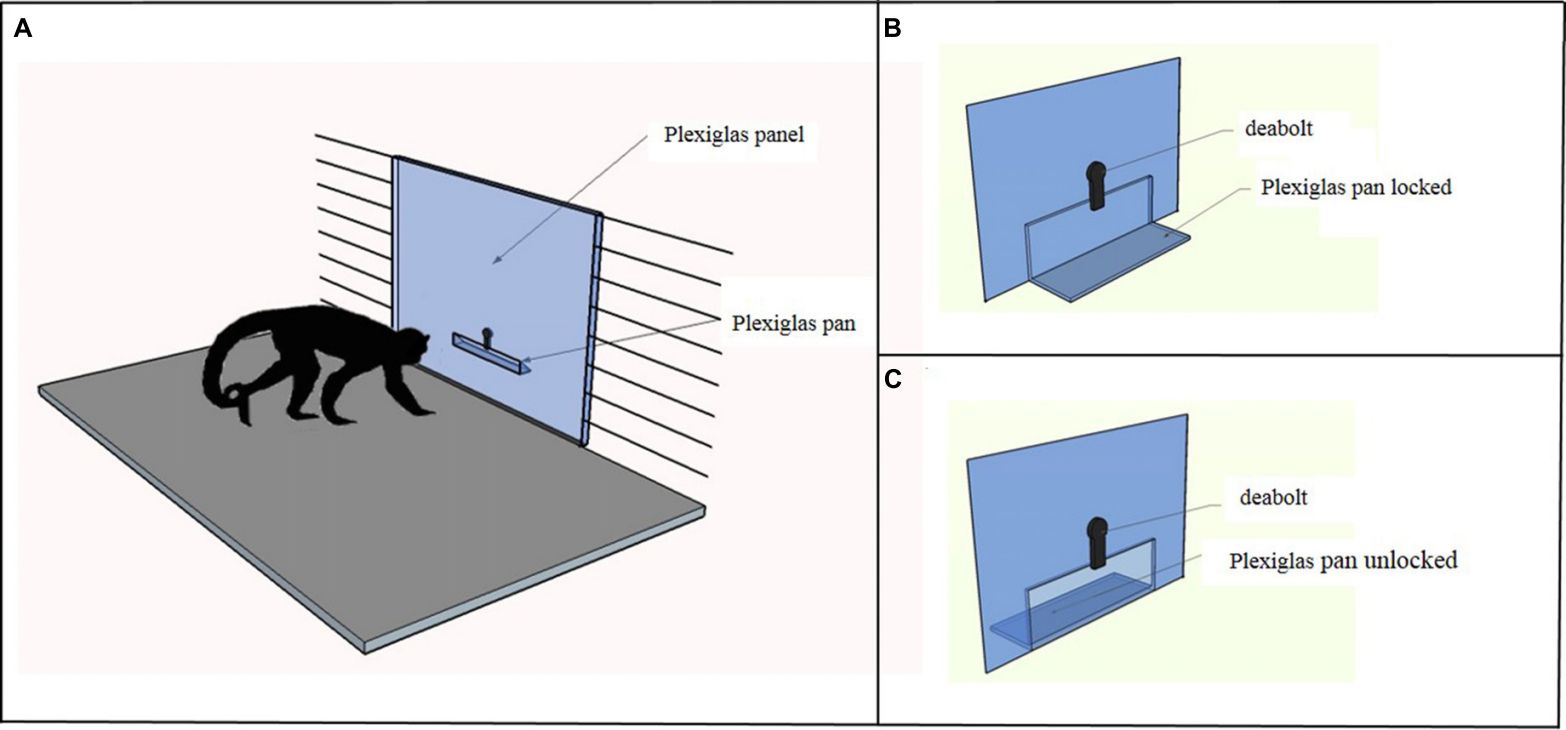
**(A)** The figure depicts the apparatus used in the Accumulation task. The experimenter placed the food items in a Plexiglas pan, which could be locked with a deadbolt. **(B)** In the single forced-accumulation trial of the Accumulation task the Plexiglas pan was locked and the experimenter accumulated 10 food items before unlocking the pan and allowing the subject to take all the items. The subject could observe the accumulation process but reaching for food was prevented until the experimenter unlocked the pan. **(C)** In the three free-accumulation trials of the Accumulation task the Plexiglas pan was unlocked and the subject could have access to the food items during the accumulation process.

Capuchins were tested in a testing box measuring 180 cm × 75 cm × 75 cm. As shown in **Figure 1A**, in the Accumulation task the apparatus was a vertical Plexiglas panel (56.6 cm × 74 cm) inserted in place of one of the three vertical mesh walls of the testing box. A Plexiglas pan (25 cm × 6.5 cm), in which the food items were placed, was attached on the experimenter’s side at 14.5 cm from the bottom of the panel. The experimenter could either lock (**Figure 1B**) or unlock (**Figure 1C**) the pan by sliding a deadbolt; when it was unlocked, capuchins could pull the pan into their side of the panel.

**FIGURE 2:**
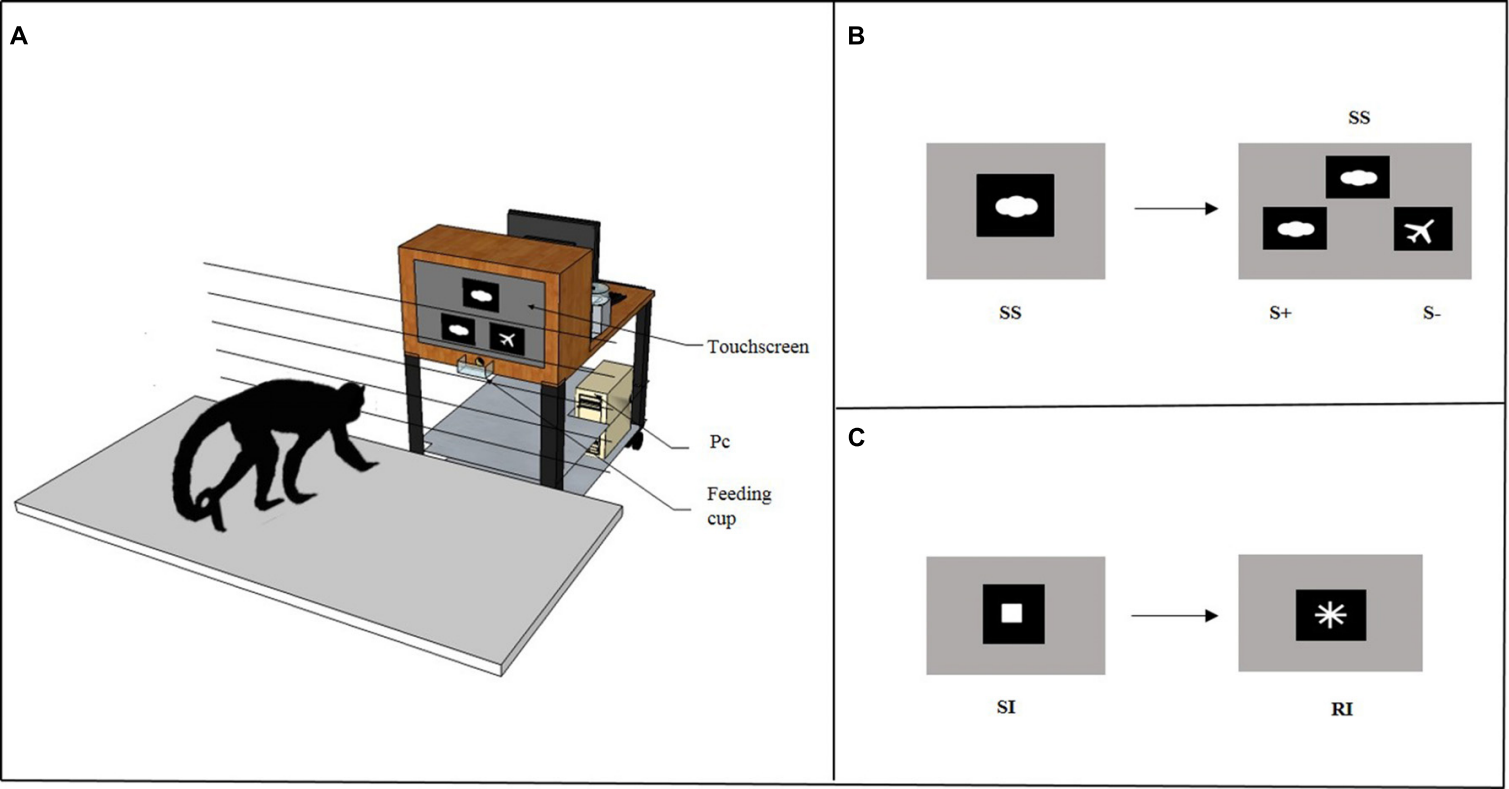
**(A)** The figure depicts the apparatus used in the Cognitive Depletion Experiment. It consisted in a computerized system mounted on the top shelf of a trolley connected to a 19″ touchscreen and to an automatic food dispenser. When the subject provided a correct response, the food dispenser delivered one banana-flavored pellet as reward into a Plexiglas feeding cup. **(B)** *High Cognitive Depletion/Identity Matching-to-Sample task*. At the beginning of each trial, an image was presented as a sample stimulus (SS) on the upper central half of the screen. When the subject touched the SS, two comparison stimuli were immediately displayed on the right and on the left, below the sample. The comparison stimuli included one matching stimulus (S+), identical to SS, and one non-matching stimulus (S-), different from SS. **(C)** *Low Cognitive Depletion/Simple Touching task*. At the beginning of each trial a white square was presented as a starting image (SI) in the center of a black background screen. When the subject touched the SI, it vanished and was immediately replaced by a rewarding image (RI), a white cross.

In the Cognitive Depletion Experiment (see below), before the Accumulation task subjects solved either a Simple Touching task or an Identity Matching-to-Sample task through a computer connected to a touchscreen. As shown in **Figure 2A**, the computerized system consisted of a personal computer (Model AMD Athlon 1200) connected to a 19″ touchscreen (Model E96f+SB, CRT, ViewSonic) and to an automatic food dispenser (Model ENV-203-45, MED Associates, Inc. Georgia, VT, USA). The E-Prime software (Psychology Software Tools, Inc.) was used as the stimulus generator and served both to present the stimuli and to record the response behavior. The food dispenser was designed to deliver one 45-mg banana-flavored pellet (TestDiet, Richmond, IN, USA) when the monkey provided a correct response during the experimental trial. The pellet was delivered into a Plexiglas feeding cup (10 cm wide × 5 cm deep × 3.5 cm high) located 16 cm below the touchscreen in the center. A wooden frame (48 cm wide × 64 cm high × 30 cm deep) with a central aperture (36 cm wide × 26 cm high) surrounded the touchscreen. The food dispenser was placed behind the wooden frame, out of sight of the subject. Moreover, an additional LCD monitor was placed at the back of the touchscreen to allow the experimenter to remove the apparatus at the end of the session. The touchscreen, food dispenser and additional LCD monitor were mounted on the top shelf of a trolley (81 cm long × 45 cm wide × 80 cm high), whereas the personal computer was on the bottom shelf. The apparatus was placed 15 cm from the grid of the testing box within the arm’s reach of the monkey. The grid was made of horizontal metal bars (0.5 cm thick) that were separated by 4.5 cm.

### Study Design and Procedure

All subjects were tested first in the Cognitive Depletion Experiment and then in the Energy Depletion Experiment. Each experiment involved three phases: (i) *pre-test baseline*, (ii) experimental phase (involving two conditions for each experiment, whose order of presentation was counterbalanced across subjects and experiments), and (iii) *post-test baseline*. In each phase, subjects were tested for 5 days (for a total of 20 days, since the experimental phase included two conditions). On each day, subjects were tested in the Accumulation task, involving four trials: (i) one forced-accumulation trial for familiarization (presented at the beginning of the session), in which the Plexiglas pan was locked and the experimenter accumulated 10 food items before unlocking the pan and allowing the capuchin to take all the items; (ii) three free-accumulation trials, in which the Plexiglas pan was unlocked and the capuchin had access to the accumulating items throughout the accumulation process. In all trials a 2-s accumulation rate was employed. Each trial ended when the subject put the last piece of food into the mouth. As food items, capuchins received small pieces of peanut (about 1/8 of a peanut seed, each weighing on average 0.11 g). There was a fixed intertrial interval of 30 s. Theoretically, if intertrial intervals are fixed regardless of subject’s choices, the subject may prefer to be impulsive to proceed more quickly to the next trial. Although there is still debate on this issue, at least in the Delay choice task even well-trained non-human animals belonging to different species (including non-human primates) do not pay attention to uncued intertrial intervals possibly because they have difficulties in learning about events that follow a reward, rather than about those which predict or precede it (reviewed in Hayden, 2015).

In the *pre*- and *post-test baseline*, on each day capuchins were tested only in the Accumulation task. These phases aimed to evaluate whether capuchins’ performance in this self-control task varied in the course of the study. In the experimental phases of both experiments, on each day capuchins were tested in the Accumulation task after the corresponding self-control depletion manipulation (see below).

#### Energy Depletion Experiment

In this experiment, capuchins were tested in the Accumulation task after their main meal (*Low Energy Depletion* condition) or right before receiving it (*High Energy Depletion* condition). To ensure that the subjects’ initial energetic state was as much as possible the same in the two experimental conditions, capuchins were tested always at the same time of the day, in the early afternoon after about 24 h from having received their daily meal. Although during the day they were tested in other experiments (and in some of them received small food rewards), this is the time of the day in which they were most likely energy depleted. Given that capuchins have a rather fast gut transit time (ranging from 1.75 to 3.5 h; Milton, 1984; Lambert, 1998; Wheeler and Tiddi, unpublished data, cited in Wheeler et al., 2013) and an estimated gastric emptying time of 2 h (Janson and Vogel, 2006), it is reasonable to suppose that they were hungry when tested in the *High Energy Depletion* condition.

##### Low energy depletion

Before being tested in the Accumulation task, the experimental subject was separated from the group in the indoor enclosure and received its carbohydrate-rich main meal, composed of 250 g of apple, 50 g of carrot, 60 g of banana and 60 g of bread. The food was located in a plastic container that the experimenter kept close to the cage to allow the animal to easily take it. Subjects were tested in the Accumulation task after 30 min from the beginning of the consumption of the meal because in humans glucose level begins to rise about 10 min after ingesting foods rich in carbohydrates (American Diabetes Association, 2001), especially if they have a high glycemic index (as for example banana and bread, two of the foods provided to capuchins in the present study). Moreover, this is the time required to reach a sense of satiety (Davis and Smith, 1990).

##### High energy depletion

Subjects were tested in the Accumulation task right before receiving their main meal, thus they were likely in a low energetic state.

#### Cognitive Depletion Experiment

Before the Accumulation task, subjects were tested either in a Simple Touching task (*Low Cognitive Depletion* condition, **Figure 2B**) or in an Identity Matching-to-Sample task (*High Cognitive Depletion* condition, **Figure 2C**).

##### Low cognitive depletion/Simple Touching task

In this task, at the beginning of each trial a white square (1 cm × 1 cm) on a black background was presented as a starting image (SI) in the center of the screen. When the subject touched the SI, it vanished and was immediately replaced by a rewarding image (RI), a white cross (3 cm × 3 cm) on a black background. The subject had to touch the RI to get the reward (one 45-mg banana flavored pellet). In each session a total of 100 trials was presented with an intertrial interval (ITI) of 10 s. This task did not require a high level of attention since the response behavior consisted of two consecutive simple touching actions in which discrimination processes were not involved. As soon as the subject completed the Simple Touching task, the Accumulation task began.

##### High cognitive depletion/Identity Matching-to-Sample task

In this task, at the beginning of each trial an image was presented as a sample stimulus (SS) on the upper half of the screen, in the center. When the subject touched the SS, two comparison stimuli were immediately displayed 4 cm below the sample, to the right and left, at a distance of 5 cm apart. The comparison stimuli included one matching stimulus (S+) identical to SS and one non-matching stimulus (S-) different from SS. The subject had to touch S+ to get the reward (one 45-mg banana flavored pellet). If S- was selected, no pellet was dispensed. A correct response was followed by a 5-s ITI, whereas an incorrect response was followed by both a 10-s time-out and a 5-s ITI. Each comparison stimulus was presented an equal number of times on both the right and the left position. The trials were presented in a random order. The stimulus set included 200 computer icons (which comprised both color as well as black and white shapes), presented in 100 pairs such that the two figures that formed a pair had the same color/s. Each figure was on average 3 cm × 3 cm and was presented within a black frame (6.5 cm × 6.5 cm).

As in the previous condition, two touching actions were required to obtain the food reward (one to SS and one to S+); however the Identity Matching-to-Sample task required a higher level of attention since the response behavior involved both discrimination processes to match stimuli that were physically identical and the ability to choose following an identity concept (for a review see, for example, Katz et al., 2007). We adopted the Identity Matching-to-Sample task since all capuchins could solve the task significantly above the chance level although without achieving a ceiling effect. As soon as the subject completed the Identity Matching-to-Sample task, the Accumulation task began.

To ensure that the subjects’ energetic state was as much as possible the same in the two experimental conditions, in Experiment 1 capuchins were tested always at the same time of the day (in the early morning before being tested in other experiments). Moreover, at the end of the Identity Matching-to-Sample task and before the Accumulation task, they received the exact number of pellets not obtained because of the errors made during the session. This allowed to equalize the total amount of food obtained by the subjects in the two experimental conditions (i.e., 100 45-mg pellets).

### Statistical Analysis

To evaluate capuchins’ performance in the Accumulation task, for each experiment we fit a conditional fixed effects negative binomial regression model with the number of food units accumulated as the dependent variable, and condition (high and low depletion), session number (within the same condition), and trial number (within the same session) as independent variables. Regression methods for longitudinal data analysis account for interdependency and structuring of the data and thus allow the use of multiple data points from the same subject (rather than aggregating all measurements of the same subject into an average value and make these values the unit of analysis), while avoiding the problem of pseudo replication. These models are particularly suited for analyzing behavioral and ecological data that typically have one or more levels of aggregations (Snijders and Bosker, 1999; van de Pol and Wright, 2009). To assess whether food eaten in the *Low Energy Depletion* condition varied significantly across sessions we performed a Friedman ANOVA. We ran the analysis in Stata 11.0 and Statistica 7. Statistical significance was set at *p* ≤ 0.05.

## Results

### Energy Depletion Experiment

**FIGURE 3:**
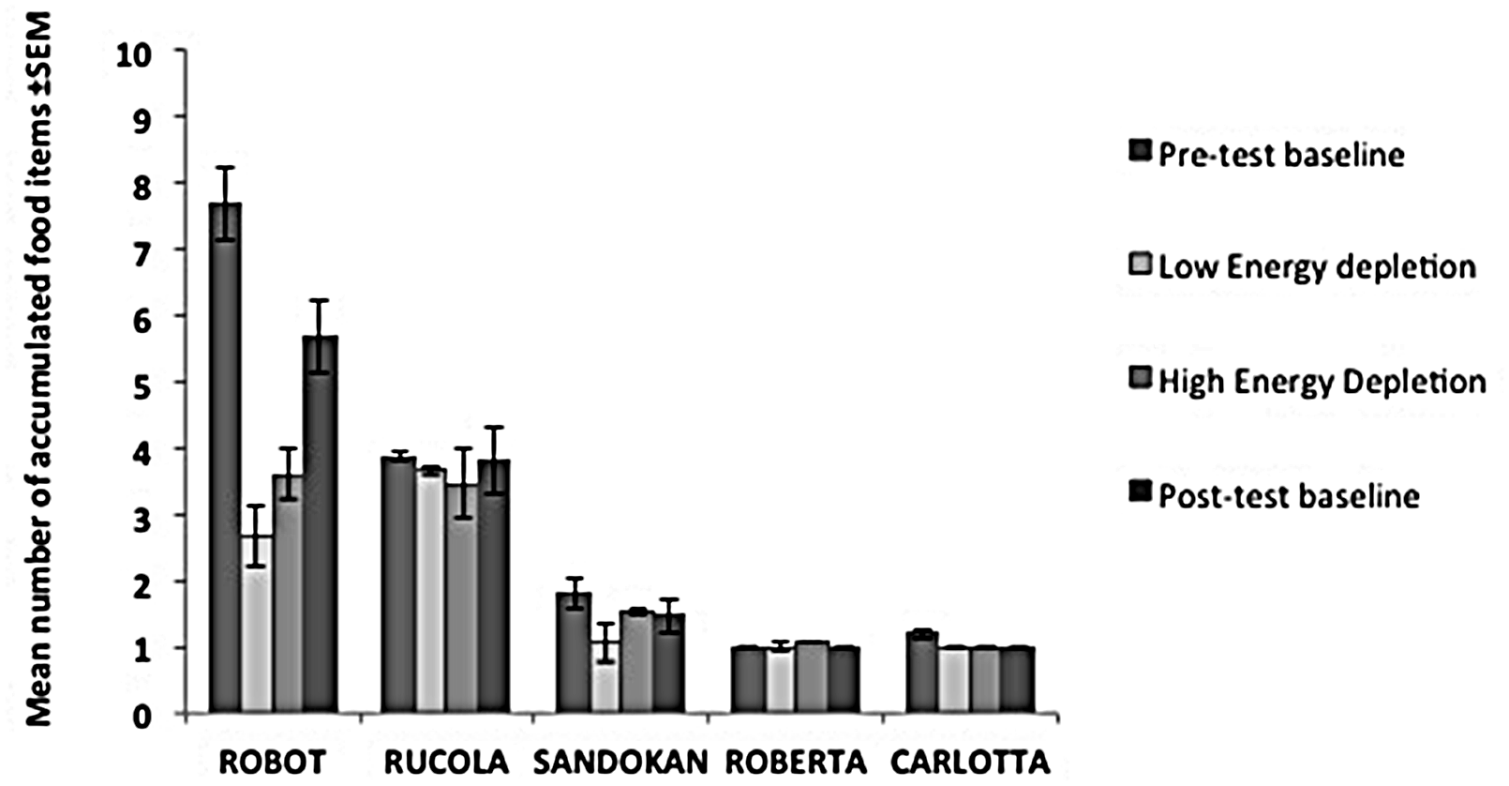
**Performance of each subject in the *Energy Depletion Experiment*.** The histograms report the mean number of food items accumulated (and SEM) for each condition in the Accumulation task.

**FIGURE 4:**
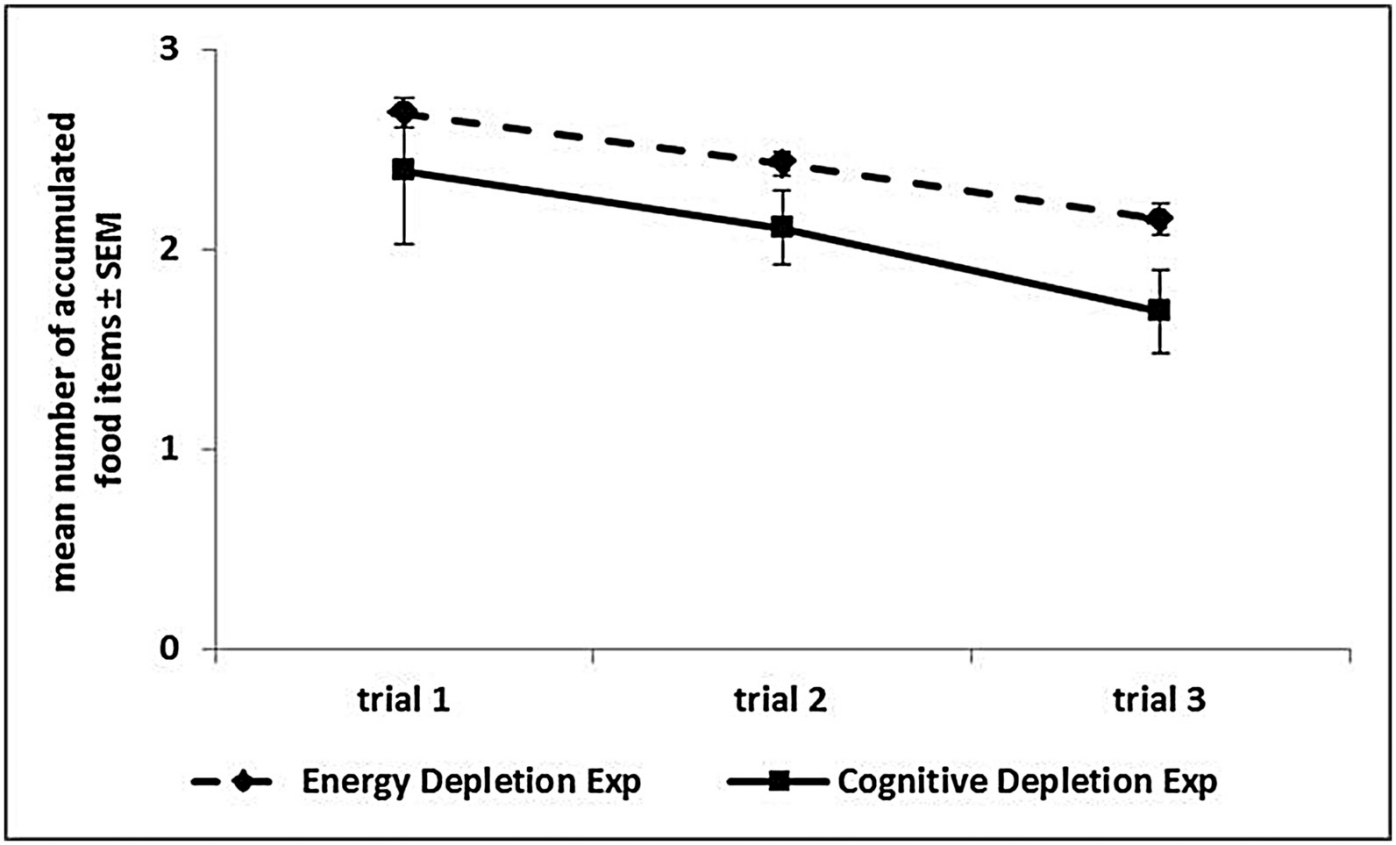
**Performance over trials in the Accumulation task.** The graph reports, for each experiment, the mean number (and SEM) of food items accumulated in each of the three trials, averaged across sessions, and experimental phases.

In the *Low Energy Depletion* condition, capuchins consumed on average 36% of the total amount of food provided (bread: 49%, bananas: 41%, apples: 30%, carrots: 23%). **Table 1** reports, per individual, the amount of food eaten, which did not significantly vary across sessions (Friedman ANOVA: *z* = 3.09, df = 4, *p* = 0.54, *N* = 4; unfortunately for one subject quantitative data on food consumption were not available). In each condition, individual performance in the Accumulation task is shown in **Figure 3**. Within-session performance significantly decreased across trials (*z* = -2.41, *p* = 0.016; **Figure 4**); moreover, there was a significant interaction between experimental condition and session number ($\chi_{3}^{2}$ = 8.88, *p* = 0.03). Across sessions, performance significantly increased in the *Post-test baseline* (*z* = 2.38, *p* = 0.017), but did not significantly vary in the other three conditions (*Pre-test baseline*: *z* = -0.51, *p* = 0.61; *High Energy Depletion*: *z* = -0.11, *p* = 0.91; *Low Energy Depletion*: *z* = -1.72, *p* = 0.09). There were no other significant interactions.

**Table 1:** Individual percentage of food eaten in the *Low Energy Depletion* condition of Experiment 1.

	Session 1	Session 2	Session 3	Session 4	Session 5
Roberta	75.0	76.2	56.2	62.5	57.5
Rucola	27.5	18.7	17.5	20.0	31.2
Robot	8.7	16.2	8.7	23.7	55.0
Sandokan	30.0	25.0	35.0	52.5	21.2

*Carlotta’s data were not available due to experimenter’s error*.

### Cognitive Depletion Experiment

In the Identity Matching-to-Sample task, at a group level capuchins showed a mean percentage of correct responses of 80.2% and their accuracy did not significantly vary across sessions (conditional fixed effects logistic regression: z = 0.86; *p* = 0.39). At the individual level, all capuchins were significantly above chance level in each session (see **Table 2**). Moreover, capuchins’ matching accuracy in the present study did not significantly differ from that observed in a previous study by Truppa et al. (2010) [this study = 80.2%; Truppa et al., 2010 = 78.2%; *t*(4) = -0.69, *p* = 0.531]. Obviously, in the Simple Touching task capuchins showed 100% of correct responses. Overall, the Simple Touching Task lasted a few minutes more than the Identity Matching-to-Sample task (mean ± SE: MTS: 17.97 ± 1.04; Simple Touching: 22.32 ± 1.60). However, considering that at the end of each Identity Matching-to-Sample task session capuchins were provided with the extra pellets corresponding to the incorrect responses (and spent time to eat them), the duration of the two tasks was approximately equivalent.

**Table 2:** Individual percentage of correct responses in the Identity Matching-to-Sample task.

	Session 1	Session 2	Session 3	Session 4	Session 5
Carlotta	73^∗∗∗^	78^∗∗∗^	82^∗∗∗^	81^∗∗∗^	73^∗∗∗^
Roberta	85^∗∗∗^	83^∗∗∗^	92^∗∗∗^	80^∗∗∗^	83^∗∗∗^
Rucola	85^∗∗∗^	90^∗∗∗^	86^∗∗∗^	79^∗∗∗^	82^∗∗∗^
Robot	74^∗∗∗^	74^∗∗∗^	83^∗∗∗^	78^∗∗∗^	83^∗∗∗^
Sandokan	67^∗∗∗^	77^∗∗∗^	83^∗∗∗^	76^∗∗∗^	79^∗∗∗^

*Binomial z scores: ^∗∗∗^p < 0.001*

**FIGURE 5:**
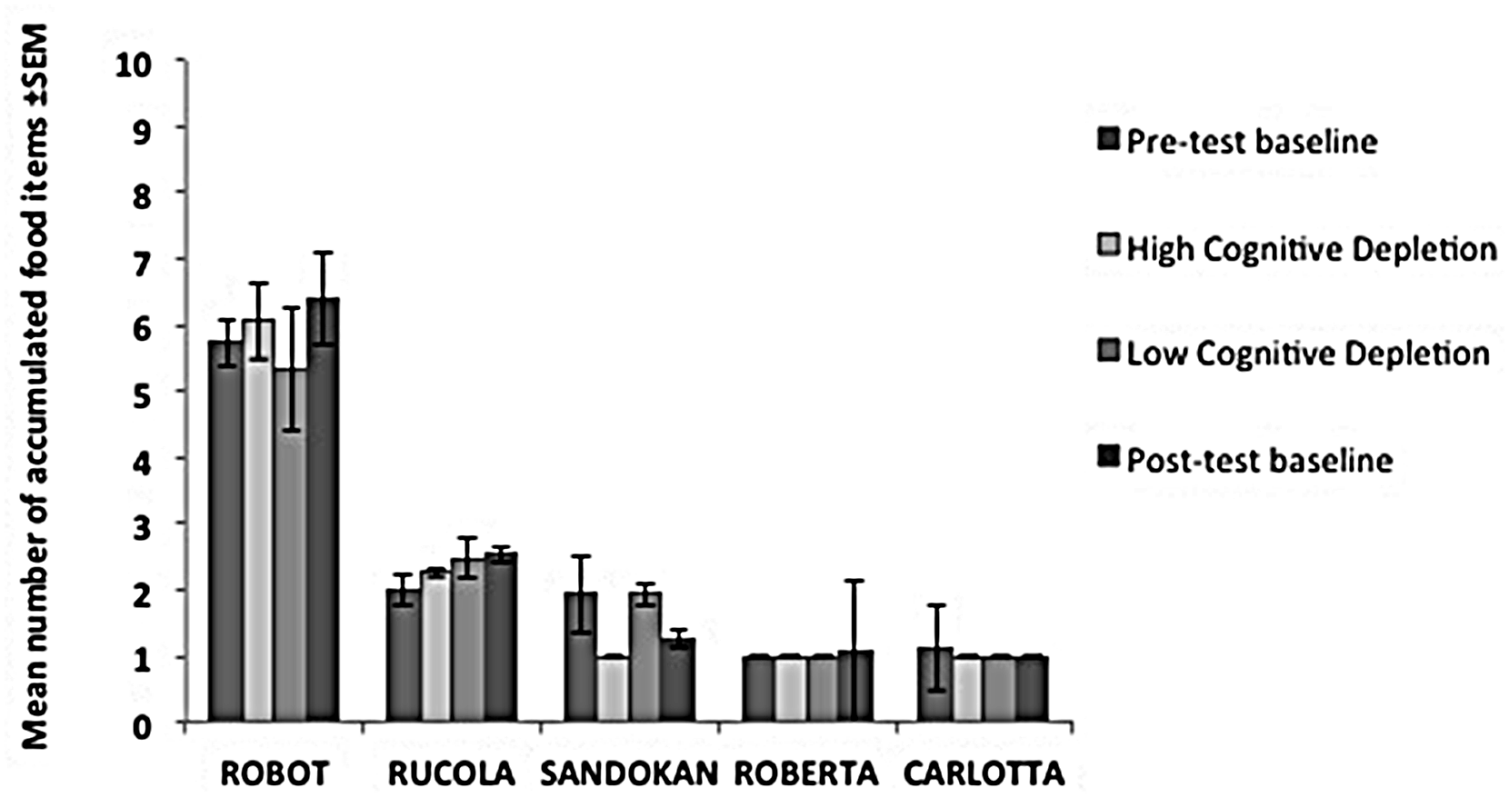
**Performance of each subject in the *Cognitive Depletion Experiment*.** The histograms report the mean number of food items accumulated (and SEM) for each condition in the Accumulation task.

In each condition, individual performance in the Accumulation task is shown in **Figure 5**. We failed to find an effect of experimental condition on capuchins’ self-control performance ($\chi_{3}^{2}$ = 0.56, *p* = 0.91). Within-session performance significantly decreased across trials (*z* = -2.76, *p* = 0.006; **Figure 4**), whereas there was no significant effect of session number (*z* = -0.56, *p* = 0.58). There were no significant interactions between experimental condition, trial, and session.

## Discussion

In the present study we aimed to test, for the first time in a non-human primate species, the Strength Model (Baumeister, 2002), by evaluating whether (i) repeated acts of self-control, (ii) energy depletion, and (iii) cognitive depletion reduce performance in a self-control task.

In both experiments capuchins’ performance in the Accumulation task decreased over trials within each session. Hence, in the short term, repeated acts of self-control in the three trials of each session progressively reduced this capacity, as predicted by the Strength Model (Baumeister, 2002). It is improbable that capuchins’ lose motivation toward food over trials because the amount of food ingested in each trial was likely not enough to induce satiety. Each subject could, in fact, accumulate a maximum of 10 items in each trial, for a total of 6.6 calories in each session, and only one subject occasionally reached this value by accumulating the maximum possible number of food items.

However, neither being tested before their main meal rather than after it (Energy Depletion Experiment), nor taking part in a highly demanding cognitive task relatively to a very simple task (Cognitive Depletion Experiment) decreased performance in a subsequent self-control task. Thus, our findings failed to confirm previous results obtained in humans (e.g., Gailliot and Baumeister, 2007; Kiesel et al., 2010; Schneider and Anderson, 2010; Barutchu et al., 2013), dogs (Miller et al., 2010, 2015; Miller and Bender, 2012), and honeybees (Mayack and Naug, 2015).

Specifically, in the Energy Depletion Experiment, capuchins were expected to accumulate a number of food items significantly lower before consuming their main meal (*High Energy Depletion* condition) than after consuming it (*Low Energy Depletion* condition). In contrast, we found that capuchins’ performance in the Accumulation task did not significantly vary in the two conditions. On the one hand, our result is in agreement with those studies showing that energy depletion does not decrease self-control performance in humans (Kurzban, 2010; Molden et al., 2012; Lange and Eggert, 2014). On the other hand, it diverges from findings indicating that low levels of blood glucose predict poor performance in self-control tasks in humans and dogs (Gailliot et al., 2007; for a review see Gailliot and Baumeister, 2007; Miller and Bender, 2012). The absence of an energy depletion effect in our study is unlikely due to an insufficient amount of food eaten by capuchins in the *Low Energy Depletion* condition since they consumed, in a brief time window, about half of their carbohydrate-rich food daily ratio, thus presumably reaching satiation. However, it cannot be excluded that capuchins were not sufficiently depleted in the *High Energy Depletion* condition, although – given their fast gut transit time (Milton, 1984; Lambert, 1998; Wheeler and Tiddi, unpublished data, cited in Wheeler et al., 2013) and gastric emptying time (Janson and Vogel, 2006) – it is reasonable to suppose that this was not the case. Future studies are needed to evaluate whether providing capuchins with a glucose solution, rather than with a carbohydrate-rich food mixture, would result in a more effective manipulation of their energetic state. Moreover, it would be important to provide capuchins with a non-sugary meal with the same caloric content as the sugary meal to disentangle the effect of satiation and blood glucose level on self-control.

In the Energy Depletion Experiment, we also found a significant interaction between experimental condition and session number. Specifically, across sessions, performance significantly increased in the *Post-test baseline*, but did not significantly vary in the other three conditions. This result is likely due to the behavior of Robot, the subject who overall accumulated the largest number of food items. His performance in the Accumulation task decreased in the *Low Energy Depletion* condition probably because of unstable social dynamics in his group in the same period of time during which our study took place. We observed, in fact, several episodes of aggression between Robot (the beta male) and Patè (the alpha male), leading to a rank reversal between them, after which, in the last condition (*Post-test baseline*), Robot’s performance improved again.

Similarly, in the Cognitive Depletion Experiment, capuchins should have accumulated a number of food items significantly lower after the Identity Matching-to-Sample task (*High Cognitive Depletion* condition) than after the Simple Touching task (*Low Cognitive Depletion* condition). In contrast, we found that capuchins’ performance in the Accumulation task did not significantly differ depending on previous task requirements, nor varied, in the long-term, across consecutive sessions. The Identity Matching-to-Sample task represents a more cognitively demanding problem than the Simple Touching task. To obtain the reward, in the former task capuchins had to follow an identity rule to choose which of two comparison figures resembled most closely a stimulus presented as sample, whereas in the latter task capuchins had to simply touch a stimulus which appeared on the screen. Learning to solve Identity Matching-to-Sample tasks according to an identity rule could be a challenging problem for non-human species (Wright et al., 1988; Wright, 1997, 2001; Galvão et al., 2005, 2008; Bodily et al., 2008, for a review see Katz et al., 2007). Specifically, our monkeys underwent a long training protocol before they succeeded in generalization tests in which they succeeded in learning to solve the task in a way that extended beyond the training stimuli, thus demonstrating to rely on relational learning processes rather than on item-specific learning (Truppa et al., 2010). Notwithstanding this, our findings indicated that the Identity-Matching-to-Sample task did not produce a significant effect ascribable to cognitive depletion in capuchins, at least when they spent about 20 min dealing with the task. It is possible that past experience with the Identity Matching-to-Sample task contributed to make it less cognitively challenging. As proposed by Baumeister and Tierney (2011), in fact, very familiar tasks do not require high levels of self-control. Indeed, we did not find a significant difference in the response accuracy when capuchins’ performance in this study was compared with that observed in their first successful generalization test (Truppa et al., 2010), nor in the present experiment capuchins’ matching accuracy varied over time. Future studies need to clarify if, and to what extent, effects due to cognitive depletion may emerge in capuchins tested in a more challenging cognitive task than the simultaneous Identity Matching-to-Sample task.

Most of the previous studies used persistence (in solving anagrams, squeezing a handgrip, and so on) to assess self-control; however, the above measure does not allow to disentangle whether a lower persistence after a potentially depleting treatment is due to a decrease in motivation or to a reduced self-control capacity (Levy, 2011; Barutchu et al., 2013). Thus, we selected a classical self-control task, the Accumulation task, a delay maintenance task that requires the subject to sustain the decision to wait for a larger or better option even if the immediate option remains available during the delay (e.g., Toner and Smith, 1977; Grosch and Neuringer, 1981; Killeen et al., 1981; Beran, 2002; Evans and Beran, 2007a,b; Pelé et al., 2011). Since the Accumulation task is a particularly challenging paradigm in which capuchins showed a lower performance compared to other species (Pelé et al., 2011; Evans et al., 2012), we cannot exclude that the lack of a significant depletion effect on capuchins’ self-control was due to task difficulty. In fact, in the present study two out of five subjects never accumulated any food item. Although our results held true also when excluding these two subjects from the sample, future studies should evaluate whether a depletion effect occurs with self-control tasks in which capuchins show a higher performance compared to the Accumulation task, such as the Delay choice task (Addessi et al., 2011, 2013, 2014). In humans, in fact, participants tested in a Delay choice task, in which they were required to choose between a smaller sooner option and a larger later option, discounted the future more after ingesting a non-caloric soft drink than after ingesting a sugary drink (Wang and Dvorak, 2010).

## Conclusion

In both experiments we found a decrease in capuchins’ self-control performance over the three trials of each experimental session. This finding may be due to a reduction of self-control caused by its repeated implementation, as predicted by the Strength Model (Baumeister, 2002), but a better understanding of the mechanism underlying this phenomenon is needed. Nonetheless, in contrast to the predictions of the Strength Model and to our initial hypotheses, consuming a carbohydrate meal did not improve capuchin monkeys’ performance in a delay maintenance task (Energy Depletion Experiment) nor being tested in a cognitively demanding task reduced their self-control ability (Cognitive Depletion Experiment). Although these results are in line with the growing body of studies that failed to find a depletion effect in humans (Kurzban, 2010; Molden et al., 2012; Carter and McCullough, 2014; Lange and Eggert, 2014; Carter et al., 2015), it cannot be excluded that our experimental manipulations were not effective enough to lead to positive findings. This was a first attempt to evaluate how energy and cognitive depletion affects self-control in a non-human primate species. Since from our results it was not possible to draw definitive conclusions, further studies are strongly needed to evaluate whether different experimental manipulations would lead to positive findings supporting the Strength Model. This is a matter for future research.

## Author Contributions

Conceived and designed the experiments: DA, EA, FDP, VT. Performed the experiments: AM, EG, FDP, VT. Analyzed the data: AM, EA, EG, FDP, VT. Wrote the paper: AM, DA, EA, EG, FDP, VT.

## Conflict of Interest Statement

The authors declare that the research was conducted in the absence of any commercial or financial relationships that could be construed as a potential conflict of interest.

## Footnotes

1 Capuchin monkeys, traditionally identified as the single genus *Cebus*, are now classified as two genera: (i) the robust (tufted) forms are classified as the genus *Sapajus*, and (ii) the gracile (untufted) forms are retained as the genus *Cebus* (Lynch Alfaro et al., 2012). Tufted capuchin monkeys hosted at the Primate Center of the CNR are derived from individuals of different provenience and are considered to be unknown combinations of species of the genus *Sapajus*.
